# Interfacing a parallel simulation of a neuronal network to robotic hardware using MUSIC, with application to real-time figure-ground segregation

**DOI:** 10.1186/1471-2202-12-S1-P78

**Published:** 2011-07-18

**Authors:** Ali Nazem, Gert Kootstra, Danica Kragic, Mikael Djurfeldt

**Affiliations:** 1 CVAP, CSC, KTH, 100 44 Stockholm, Sweden; 2 PDC, CSC, KTH, 100 44 Stockholm, Sweden; 3 INCF, Karolinska Institutet, Nobels väg 15A, 171 77 Stockholm, Sweden

## 

MUSIC, the multi-simulation coordinator, supports communication between neuronal-network simulators, or other (parallel) applications, running in a cluster super-computer [1,4]. Here, we've developed a MUSIC-enabled class library providing an interface between MUSIC-enabled applications and applications running on computers outside of the cluster. Specifically, we have used this component to interface the cameras of a robotic head to a neuronal-network simulation running on a Blue Gene/L supercomputer [2]. The interface enables the neuronal-network simulator to receive real-world images in real-time from the robot. Moreover, it enables the robot to be controlled by the neuronal network. The neuronal-network simulation implements a model of figure-ground segregation based on neuronal activity in the Macaque visual cortex [3].

I. A special purpose TCP/IP based communication interface has been implemented in C++ as an extendible class library. The architecture of the interface is shown in Figure 1. The client end of the interface is specifically designed to meet the requirements for operating on the Blue Gene /L, as well as being MUSIC-aware. The server side component resides in the outside world providing the client with streaming real-time data. We designed an inheritable class called CSerializable that defines the unit of data and marshals the data across the route from the source to the final destination in the parallel application. Based on CSerializable, we implemented the entities CCommand and CRawImage which are required for transmission of control commands and images, respectively, between the parallel application and the robot (Figure 1). A single process application called MusicGate, connects the client side of the interface and the music-enabled component together. The parallel application sends a command to the server requesting a stream of images. As soon as one frame of the data sent from the server is available in MusicGate, it will be directly transferred from the read buffer to the parallel application by the MUSIC library, hence avoiding redundant internal memory operations. The implemented architecture performs the communication IO operations in parallel with the neural processing in order to minimize the idle time in compute nodes. The idle time is a function of communication latency, and the processing load of the neuronal-network simulation.

II. Having the communication interface, we implemented a parallel model for figure-ground segregation. The implementation covers the structure of neurons, neuronal layers, and neuronal networks that can operate on a parallel platform. We defined the concept of a *tile* of neurons. One dedicated processor is allocated to each tile of neurons. Each layer contains a number of tiles.

## Results

We have successfully implemented an interface between a neuronal-network simulator running on a parallel computer and a robot in the real world. The latency and transfer rate of the entire model makes real-time figure-ground segregation possible. Since the client side of the interface provides a standard MUSIC port towards the parallel application, it can be used to connect the robotic head to a generic MUSIC-enabled neuronal-network simulator. We demonstrate this for the NEST simulator [4].

**Figure 1:**
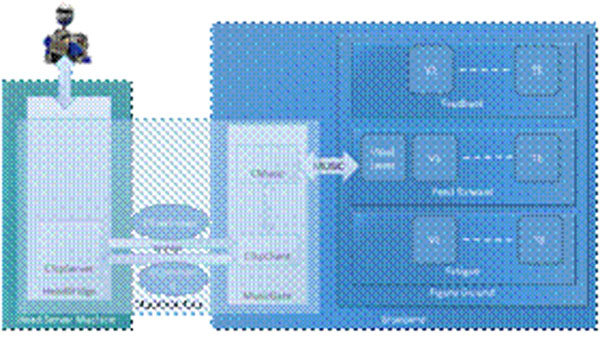
*The MUSIC interface architecture.*
